# A computational pipeline for quantification of pulmonary infections in small animal models using serial PET-CT imaging

**DOI:** 10.1186/2191-219X-3-55

**Published:** 2013-07-23

**Authors:** Ulas Bagci, Brent Foster, Kirsten Miller-Jaster, Brian Luna, Bappaditya Dey, William R Bishai, Colleen B Jonsson, Sanjay Jain, Daniel J Mollura

**Affiliations:** 1 Center for Infectious Disease Imaging, National Institutes of Health, Bethesda, MD 20892, USA; 2 Radiology and Imaging Sciences, National Institutes of Health, Bethesda, MD 20892, USA; 3 Center for Tuberculosis Research, Johns Hopkins University School of Medicine, Baltimore, MD 21231, USA; 4 KwaZulu-Natal Research Institute for TB and HIV, Durban, South Africa; 5 Howard Hughes Medical Institute, Chevy Chase, MD, USA; 6 Department of Microbiology and Immunology, University of Louisville, Louisville, KY 40202, USA; 7 The Center for Predictive Medicine for Biodefense and Emerging Infectious Diseases, University of Louisville, Louisville, KY 40202, USA; 8 Department of Medicine, Center for Infection and Inflammation Imaging Research, Johns Hopkins University School of Medicine, Baltimore, Maryland 21287, USA; 9 Department of Pediatrics, Johns Hopkins University School of Medicine, Baltimore, MD 21231, USA

**Keywords:** Quantitative analysis, Pulmonary infections, Small animal models, PET-CT, Image segmentation, H1N1, Tuberculosis

## Abstract

**Background:**

Infectious diseases are the second leading cause of death worldwide. In order to better understand and treat them, an accurate evaluation using multi-modal imaging techniques for anatomical and functional characterizations is needed. For non-invasive imaging techniques such as computed tomography (CT), magnetic resonance imaging (MRI), and positron emission tomography (PET), there have been many engineering improvements that have significantly enhanced the resolution and contrast of the images, but there are still insufficient computational algorithms available for researchers to use when accurately quantifying imaging data from anatomical structures and functional biological processes. Since the development of such tools may potentially translate basic research into the clinic, this study focuses on the development of a quantitative and qualitative image analysis platform that provides a computational radiology perspective for pulmonary infections in small animal models. Specifically, we designed (a) a fast and robust automated and semi-automated image analysis platform and a quantification tool that can facilitate accurate diagnostic measurements of pulmonary lesions as well as volumetric measurements of anatomical structures, and incorporated (b) an image registration pipeline to our proposed framework for volumetric comparison of serial scans. This is an important investigational tool for small animal infectious disease models that can help advance researchers’ understanding of infectious diseases.

**Methods:**

We tested the utility of our proposed methodology by using sequentially acquired CT and PET images of rabbit, ferret, and mouse models with respiratory infections of *Mycobacterium tuberculosis* (TB), H1N1 flu virus, and an aerosolized respiratory pathogen (necrotic TB) for a total of 92, 44, and 24 scans for the respective studies with half of the scans from CT and the other half from PET. Institutional Administrative Panel on Laboratory Animal Care approvals were obtained prior to conducting this research. First, the proposed computational framework registered PET and CT images to provide spatial correspondences between images. Second, the lungs from the CT scans were segmented using an interactive region growing (IRG) segmentation algorithm with mathematical morphology operations to avoid false positive (FP) uptake in PET images. Finally, we segmented significant radiotracer uptake from the PET images in lung regions determined from CT and computed metabolic volumes of the significant uptake. All segmentation processes were compared with expert radiologists’ delineations (ground truths). Metabolic and gross volume of lesions were automatically computed with the segmentation processes using PET and CT images, and percentage changes in those volumes over time were calculated. *(Continued on next page)(Continued from previous page)* Standardized uptake value (SUV) analysis from PET images was conducted as a complementary quantitative metric for disease severity assessment. Thus, severity and extent of pulmonary lesions were examined through both PET and CT images using the aforementioned quantification metrics outputted from the proposed framework.

**Results:**

Each animal study was evaluated within the same subject class, and all steps of the proposed methodology were evaluated separately. We quantified the accuracy of the proposed algorithm with respect to the state-of-the-art segmentation algorithms. For evaluation of the segmentation results, dice similarity coefficient (DSC) as an overlap measure and Haussdorf distance as a shape dissimilarity measure were used. Significant correlations regarding the estimated lesion volumes were obtained both in CT and PET images with respect to the ground truths (*R*^2^=0.8922,*p*<0.01 and *R*^2^=0.8664,*p*<0.01, respectively). The segmentation accuracy (DSC (%)) was 93.4±4.5*%* for normal lung CT scans and 86.0±7.1*%* for pathological lung CT scans. Experiments showed excellent agreements (all above 85%) with expert evaluations for both structural and functional imaging modalities. Apart from quantitative analysis of each animal, we also qualitatively showed how metabolic volumes were changing over time by examining serial PET/CT scans. Evaluation of the registration processes was based on precisely defined anatomical landmark points by expert clinicians. An average of 2.66, 3.93, and 2.52 mm errors was found in rabbit, ferret, and mouse data (all within the resolution limits), respectively. Quantitative results obtained from the proposed methodology were visually related to the progress and severity of the pulmonary infections as verified by the participating radiologists. Moreover, we demonstrated that lesions due to the infections were metabolically active and appeared multi-focal in nature, and we observed similar patterns in the CT images as well. Consolidation and ground glass opacity were the main abnormal imaging patterns and consistently appeared in all CT images. We also found that the gross and metabolic lesion volume percentage follow the same trend as the SUV-based evaluation in the longitudinal analysis.

**Conclusions:**

We explored the feasibility of using PET and CT imaging modalities in three distinct small animal models for two diverse pulmonary infections. We concluded from the clinical findings, derived from the proposed computational pipeline, that PET-CT imaging is an invaluable hybrid modality for tracking pulmonary infections longitudinally in small animals and has great potential to become routinely used in clinics. Our proposed methodology showed that automated computed-aided lesion detection and quantification of pulmonary infections in small animal models are efficient and accurate as compared to the clinical standard of manual and semi-automated approaches. Automated analysis of images in pre-clinical applications can increase the efficiency and quality of pre-clinical findings that ultimately inform downstream experimental design in human clinical studies; this innovation will allow researchers and clinicians to more effectively allocate study resources with respect to research demands without compromising accuracy.

## Background

There has been significant progress in the use of non-invasive imaging technologies in human (clinical) and animal (pre-clinical) research, using positron emission tomography (PET), computed tomography (CT), and magnetic resonance imaging (MRI). In addition to being used in the clinical environment on human patients, such as for diagnosing and tracking disease, CT and MRI have been used extensively in small animal research for visualization of normal and abnormal anatomical structures. On the other hand, PET imaging provides functional imaging of the biologic processes being studied, such as for the measurement of the inflammatory response in the lungs to an infectious disease or for quantifying the severity of a cancerous tumor via the radio-labeled glucose analog, ^18^F-fluorodeoxyglucose (FDG) [1,2]. Recently, the use of PET along with CT and MR imaging has been an active research area for small animal studies as well as for human studies, but automated computer-assisted tools for image analysis in small animal models are still scarce due to limited resolution, similar visual appearances between normal and abnormal adjacent tissues, and heterogeneous imaging parameters such as animal positioning, radiotracer dose, and respiratory motion. In particular, automated quantification of functional imaging data in small animal models has been very limited.

Much of the small animal model literature has relied on manual or semi-automated methods for image analysis with qualitative and/or semi-quantitative measurements [3-10]. In these (mostly manual) approaches, investigators need to visually select and then manually draw regions of interest in the images from which they extract quantifiable information. For instance, it was shown in [3] that brain tumors were precisely delineated by using a new triple imaging modality called MRI photoacoustic-Raman nanoparticle, which helped to identify brain tumor margins. This non-invasive process allowed surgeons to remove brain tumors from mice with great accuracy. In [4], lung cancers were studied in mice models, and their subsequent responses to therapy *in situ* were examined using MRI. The authors presented quantitative and analytical methods to better visualize, understand, and quantify primary and metastatic lung tumors’ severity and progression. In another study, [5], genetically engineered mouse models with non-small-cell lung cancer were used to question the molecular complexity of mixed therapeutic response. In [6], the total lung activity of mice with chemically induced lung squamous cell carcinoma was used to measure tumor metabolic activity in lesions using longitudinal PET scans. All of these approaches are highly time-consuming, and thus, reduce the efficiency of the research, in addition to lowering reproducibility and findings.

Robust, accurate, and fast analytical tools for imaging are especially needed for *infectious disease research* because infected lesions often have a rapid progression over days or weeks with heterogeneous structure (size, shapes, and locations), in addition to being anatomically multi-focal, with asynchronous changes over time. Most of the small animal studies using non-invasive imaging techniques in the literature, including the ones highlighted above, are extensively focused on cancer for measuring neoplasms.

Despite unique clinical challenges in quantification and interpretation of infectious lung diseases, researchers need to develop small animal models of infectious diseases in order to study immunopathogenesis, enhance clinical diagnostic accuracy, and test possible treatment strategies [1,11,12]. Like cancer, research focusing on infectious disease models may benefit from current functional and structural non-invasive imaging techniques, too. For example, by using a PET-CT imaging technique, one can non-invasively visualize the quality and quantity of inflammatory cell migration and aggregation, as well as kinetics over time while also obtaining immediate information about disease burden without requiring sacrifices. Primarily, longitudinal imaging studies are invaluable for analysis of infectious disease models and tracking rapid changes in immune response.

In the presented study, we first showed the feasibility of using PET-CT imaging to track pulmonary infections in small animal images. Then, we examined the relationship between the severity and extent of pulmonary infections as assessed by PET-CT imaging longitudinally. For this purpose, we created a novel computational pipeline for reliable and accurate quantification of small animal models with respiratory infections. Our study used an automated method for measuring areas of abnormal uptake on PET images and an interactive method for analyzing corresponding anatomical structures on CT images. In addition, we longitudinally assessed lesion volumes qualitatively and quantitatively in order to increase the *efficiency* and *quality* of molecular imaging studies. This proposed methodology aims to transform imaging data into a common platform so that clinicians can quantify, diagnose, and characterize disease progression readily.

### Fundamentals of PET-CT imaging

To obtain metabolic/functional information of tissues through PET scans, molecular imaging probes, such as ^18^F-FDG and NaF, are used to interrogate specific targets such as cell surface receptors, enzymes, and structural proteins [2]. Given the low resolution in PET [13], the superior anatomic localization of a lesion is achieved by fusing the PET images to CT images such that the lesions identified on PET are then anatomically localized by analyzing the corresponding cross-sectional CT slices. To obtain anatomical and physiological information from tissues and organs, CT is usually used in small animal studies since it is the gold standard for clinical practice, particularly for lung studies [3,4]. This dual-imaging modality approach provides a better understanding of the underlying disease by fusing both modalities into a single view.

#### What to measure in CT

Structural imaging methodologies (i.e., CT and MRI) provide detailed knowledge of anatomical structures such as their shape, numbers, dimensions, surfaces, geometric arrangements, locations, and relative positioning. Among these morphological measurements, the total lung volume and the fraction of lungs occupied by disease are common measurements used by clinicians and researchers to evaluate respiratory pathology. Abnormal CT imaging patterns (volume occupied by gas, tissue, and total number of alveoli) are also conventional measures frequently used by clinicians to evaluate disease state, severity, and progression of respiratory disorders, however, accurate, robust, and fast computation of these volumetric measurements require computer-aided lesion detection, image segmentation, and automatic quantification methods. Due to significant limitations in imaging (i.e., low specificity and similar appearances between normal and abnormal tissue), manual processing and computing the aforementioned metrics are still too time-consuming and difficult.

#### What to measure in PET

PET imaging, as a functional imaging methodology, provides a way for making *in vivo* measurements of specific biochemical reactions [5]. Conventionally, the standardized uptake value (SUV), a quantitative measure of tissue activity, is widely used in assessing PET images. SUV can be used either voxel-wise or over a region/volume, and particularly in the latter case, precise identification of the region of interest (delineation) plays a vital role in diagnostic decision systems. In addition, similar to the morphological metrics used in structural imaging methodologies, volume and area of activity regions, as well as its SUV-related indexes, are used to evaluate disease extent, characterization, and severity. In other words, the precise volume/surface information of uptake regions is needed due to two reasons: (a) total volume/surface occupied by radiotracer activity can be used independently to compare the fraction of the lung affected by the infection to the fraction of the abnormal anatomical structure having activity, because only a small percentage of the abnormal tissues (i.e., consolidation) may have high metabolic activity, depending on the disease pathology, and (b) the accurate computation of SUV-related evaluation metrics requires precise delineation of uptake regions from PET scans. Even small errors in delineation can distort SUV calculations by changing the margin of the uptake regions [6], and this can eventually affect the characterization of the disease, evaluation of response to therapy, and the therapy planning.

### Structural and functional imaging patterns pertaining to pulmonary infections

**Figure 1:**
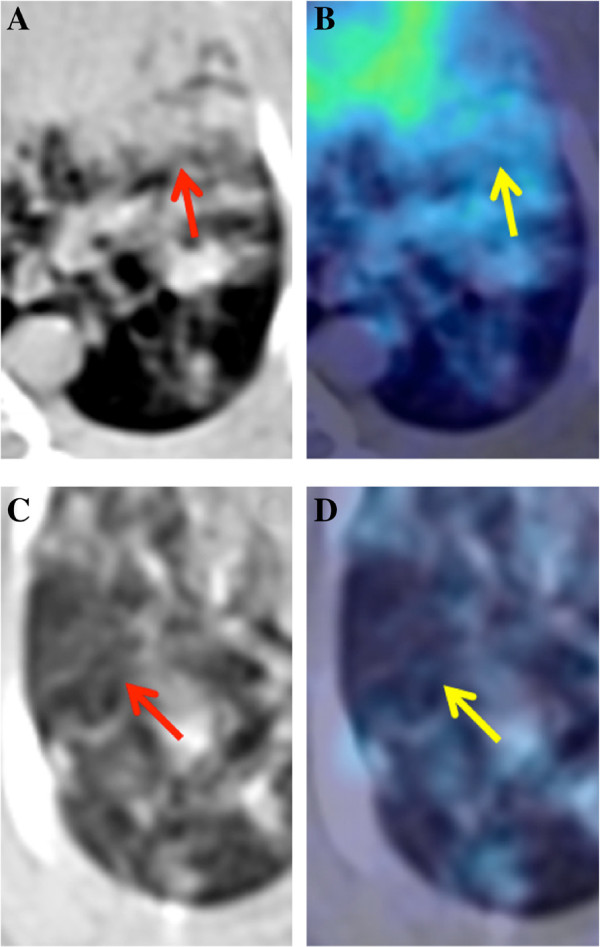
**Axial CT and fused PET-CT images from an infected rabbit lung.** Representation of two abnormal imaging patterns common to infection: arrows in the top row point **(A, B)** to a large area of diffuse consolidations, while the arrows in the bottom row **(C, D)** indicate an area of ground glass opacity (GGO).

Structural and functional abnormal imaging patterns are often observed when lungs are infected. During a CT examination, the infected lungs may include the following abnormal imaging patterns: ground glass opacities (GGO), tree-in-bud (TIB) nodularities, reticular opacities, random distribution of nodules, and consolidations [4]. Though these visual patterns are not specific for one pathogen, the proportion of lung volume exhibiting these formations can provide further insights into the severity of the infection [10]. On the other hand, a PET examination shows increased radiotracer uptake in regions of inflammation caused by infection over the background levels of uptake, in the surrounding normal tissues. Although SUV measurements are not specific to one type of infection and cannot reliably distinguish between infection, neoplasm, or inflammation, the focally increased areas of FDG radiotracer activity represent heightened cellular glucose metabolism which can be serially assessed in order to investigate the progression of a disease state. In our small animal infectious disease models, we mostly observed GGO and consolidations (shown in Figure 1) on CT, with corresponding areas of abnormal radiotracer activity on PET.

## Methods

### PET-CT imaging

The Administrative Panel on Laboratory Animal Care approvals were obtained from each participating institute prior to conducting this research. Internationally recognized standard guidelines were followed as complied with laboratory animal care approvals.

Our study included rabbits, ferrets, and mice, which are frequently used small animals in infectious disease research. Following baseline scans, ten rabbits (New Zealand White female) were infected with an aerosolized respiratory pathogen (*Mycobacterium tuberculosis*, H37Rv strain) in a Madison chamber and imaged every 5 weeks post-infection for 38 weeks or until euthanized for necropsy. For PET imaging, the rabbits were injected with 1 to 2 mCi of ^18^F-FDG PET radiotracer, via the marginal ear vein, and then imaged 45 min post-injection for a period of 30 min. CT and PET imaging were performed without respiratory gating on the NeuroLogica CereTom (NeuroLogica Corporation, Danvers, MA, USA) and the Philips Mosaic HP scanner (Philips Medical Systems, Eindhoven, The Netherlands), respectively, with specifications as reported in Tables 1 and 2. A total of 92 images (46 PET and 46 CT scans) were used for this rabbit study.

**Table 1:** Imaging parameters for ferret, rabbit, and mouse studies (CT scan)

**Animal**	**CT scanner**	**Voxel**	**In-plane**	**Current (*μ*A),**	**Slice**
		**size (mm**^**3**^**)**	**resolution**	**voltage (kVp)**	**thickness (mm)**
Rabbit	NeuroLogica CereTom (CT)	0.29 × 0.29 × 0.63	512 × 512	5,000, 120	1.25
Ferret	Siemens Inveron	0.20 × 0.20 × 0.20	384 × 384	500, 80	0.21
	Trimodal (PET/CT)				
Mouse	Mediso Bioscan Inc (CT)	0.20 × 0.20 × 0.20	176 ×176	1,500, 45	0.20
	Trimodal (PET/CT)				

**Table 2:** Imaging parameters for ferret, rabbit, and mouse studies (PET scan)

**Animal**	**PET scanner**	**Voxel**	**In-plane**	**Slice**	**Radiotracer**
		**Size (mm**^**3**^**)**	**resolution**	**thickness (mm)**	
Rabbit	Philips Mosaic HP (PET)	1.0 × 1.0 × 1.0	128 ×128	1.0	1.2 mCi ^18^F-FDG
Ferret	Siemens Inveron	0.78 × 0.78 × 0.80	128 × 128	0.8	2 mCi ^18^F-FDG
	Trimodal (PET/CT)				
Mouse	Philips Mosaic HP (PET)	0.20 × 0.20 × 0.20	128 ×128	1.0	0.2 mCi ^18^F-FDG

For the ferret study, following baseline scans pre-infection, 12 ferrets (*Mustela putorius furo*) were inoculated intra-nasally with a respiratory pathogen (the H1N1 influenza virus) and then subsequently imaged on days 1, 2, 3, and 6 post-infection or until euthanized for histopathology analyses. The ferrets were injected intra-peritoneally with 2 mCi of ^18^F-FDG and imaged 60 to 90 min post-injection. All PET and CT imaging were performed with respiratory gating on a Siemens Inveon Trimodal scanner (Siemens AG, Munich, Germany) (Tables 1 and 2). A total of 44 images (22 PET and 22 CT scans) were used for the ferret study. Although respiratory gating was used in the ferret images, ungated CT could also be used. As reported in [14], the effects of inspiration and expiration can be ignored in small animal CT scans of ferrets due to similar quality of ungated and gated CT scans.

For the mouse study, after baseline scans, three mice were infected with an aerosolized respiratory pathogen (necrotic TB) in a Glas-Col inhalation exposure chamber (Glas-Col, Terre Haute, IN, USA) and subsequently imaged on weeks 6, 10, and 14 post-infection or until euthanized for histopathology analysis. Mice were injected with approximately 0.200 mCi of ^18^F-FDG, via tail vein, and then imaged 45 min post-injection. PET images were collected using a Philips Mosaic HP scanner, and CT images were acquired using a Bioscan Inc Mediso SPECT/CT scanner (Bioscan Inc, Paris, France) (Tables 1 and 2). All images were acquired without respiratory gating. Although respiratory gating may improve image quality with less noise and motion artifacts, due to well-known effects of breathing cycle on CT imaging of mice, repeated *in vivo*-gated CT scans are recommended only not more frequently than weekly intervals [15]. However, our model was not optimized for that convention (i.e., having 4-week intervals between scans), therefore, the free-breathing mode was selected as complied with the standard imaging techniques for similar conditions in other studies. A total of 24 images (12 PET and 12 CT scans) were used for the mouse study. Details of each small animal species’ CT and PET imaging parameters, as well as scanner and reconstructed image properties, can be found in Tables 1 and 2.

Small animals were imaged in different scanners, at different institutions, with varying scanner parameters (i.e., respiratory gating, slice thickness, resolution, etc.), therefore, our study constituted a good set of images for verifying the ‘robustness’ of the proposed computational platform, in terms of handling varying conditions, as well as different animal sizes and anatomies.

### Proposed computational framework

**Figure 2:**
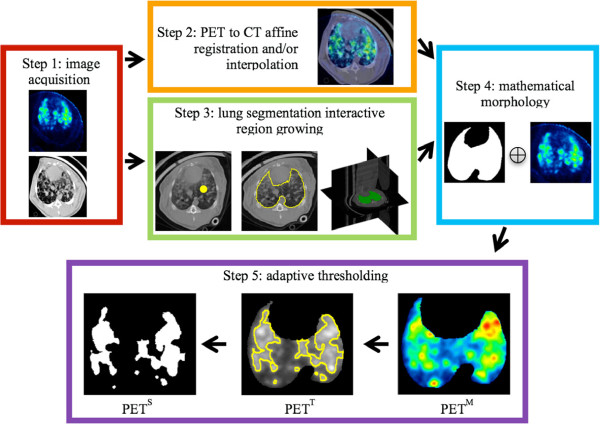
**Determination of PET functional volume in an infected rabbit.** CT and PET images are acquired from the scanner (step 1). Affine registration of images is conducted to achieve a one-to-one voxel correspondence between CT and PET images (except when the images are in registration due to simultaneous acquisition) (step 2). Lung segmentation is achieved using an interactive region growing (IRG) algorithm (step 3). The segmented lung is used as a binary mask in whole body PET imaging in order to bound functional uptake only pertaining to the lungs (PET ^M^) (step 4). Significant uptake regions of PET ^M^ are delineated using an adaptive thresholding method (step 5). Resulting segmentation of uptake regions (PET ^S^) and segmented regions with PET intensity values (PET ^T^) are used for quantification and monitoring of the disease extent.

The proposed computational framework for longitudinal and quantitative analysis of lung abnormalities is illustrated in Figure 2. The following are the steps involved in the computational framework for longitudinal and quantitative analysis: 

*Step 1.* PET and CT images were acquired from the scanner.

*Step 2.* PET and CT images were aligned into a common anatomical reference space in order to provide one-to-one voxel correspondences between anatomical and functional images (if the images were not from the same scanner). See Additional file 1 for technical details.

*Step 3.* The lungs were segmented from CT scans using the proposed interactive region growing (IRG) image segmentation method. Since measurements within the lung volumes play a significant role in identifying the nature of lung disorders, it is desirable to constrain the analysis of PET uptake regions.

*Step 4.* Mathematical morphology (smoothing by the erosion, prior to the space multiplication of two images) was conducted between the segmented lung volumes from CT scans and corresponding PET images in order to avoid FP uptake from nearby structures of the lungs. This may occasionally appear in the border of the lungs due to the resolution difference of PET and CT as well as the interpolation operator. Resultant PET images, therefore, only show radiotracer uptake regions within the lung.

*Step 5.* Significant radiotracer uptake regions were isolated from background noise and tissue through segmentation. To do this, we segmented radiotracer uptake regions from PET ^M^ images using adaptive thresholding, following clinical conventions in SUV computations as well as boundary uncertainty information from fuzzy c-means algorithm (FCM). All of these steps are explained in the following subsections in details.

#### Ground truth construction

**Figure 3:**
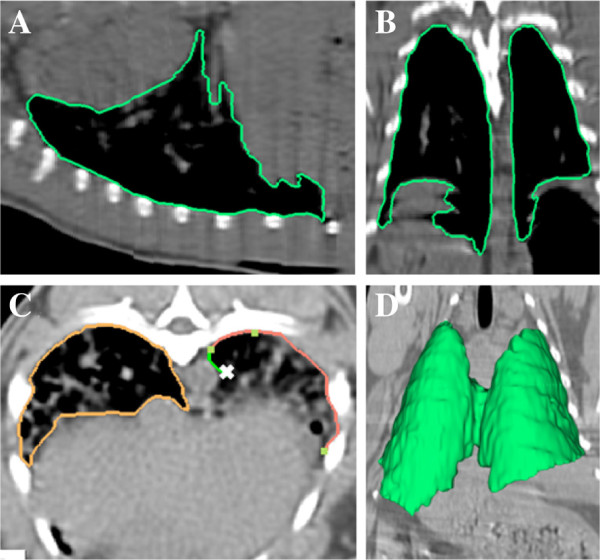
**Sample delineation process via LW.** Sagittal **(A)**, coronal **(B)**, and axial **(C)** plane from a rabbit CT scan. As seen in **(C)**, the user provides guidance by clicking a few anchor points and LW automatically traces the boundary of interest between anchor points. **(D)** 3D visualization of the segmented lungs, at the end of LW process, is shown in green.

Establishing the true delineation of any object of interest (i.e., lung region, pathological object, etc.) from biomedical images is impossible when histology images are not available [16], therefore, an appropriate surrogate truth is often used when evaluating segmentation methods. For a surrogate truth of the gold standard (namely the ground truth), participating expert radiologists used the live-wire (LW) algorithm [17,18] to segment the lungs in the sagittal, coronal, and axial domains simultaneously. In the LW algorithm, as demonstrated below in Figure 3, the user provided recognition help by tracing the boundary of the object of interest and the computer delineated the object’s boundary by automatically snapping to the edges. Even though the LW algorithm was used for ground truth construction, observers were expected to manually correct the resulting delineations in order to follow the standards of the state-of-the-art ground truth construction techniques [19,20]. For this purpose, any other manual tracing methods could also have been used, but since the LW algorithm provides a globally optimal solution for the segmentation given their respective cost functions, we preferred to use the LW method in order to guarantee that the algorithm did not to fall into local minima far from the best solution and minimize the intra- and inter-observer variations through recognition help [17,21].

To test the accuracy and efficiency of our proposed IRG and adaptive thresholding segmentation methods, we used a widely accepted evaluation criterion of DSC [16,22,23] and Haussdorf distance (HD) [23]. DSC is a measurement of spatial overlap (in percentage) between segmented object and ground truth, and HD is a shape dissimilarity metric measuring the most mismatched boundary points between the segmented object and ground truth delineation. While higher DSC and lower HD values indicate a highly accurate segmentation, one may need to also consider inter- and intra-observer agreement rates because it is often expected that the proposed method should be comparable to one of those agreement rates to determine if the method is accurate and robust. For further information on these evaluation metrics and their computations, see Additional file 1.

#### Co-registration of PET-CT scans

When fusing the complementary information obtained from different imaging modalities, there is a need for image registration to integrate anatomical context with functional image information [24]. This is particularly important in studies where PET and CT images are acquired from separate scanners or when PET and CT are done at different times, both with possible interval changes in animal positioning. In our work, image registration is treated as an optimization problem, which uses normalized mutual information as a similarity metric to align different image modalities of the same subject [25]. See Additional file 1 for further technical information on image registration used in this study.

#### Interactive image segmentation of lung volumes from CT scans

Region growing (RG) is one of the simplest, yet most effective, image segmentation approaches for segmenting lung regions within CT scans [23,26]. Segmentation via RG methods is generally favored particularly in noisy images where borders are extremely difficult to detect. Although RG has been successful with the segmentation of the normal lungs in both human and small animal studies, pathological lung tissue caused by pulmonary infections can make automatic lung segmentation from CT scans very challenging. In practice, the boundaries between dense abnormal regions such as consolidations and other organs are not well defined, therefore, we used IRG segmentation, where minimal user interaction was required for the segmentation of dense abnormal regions that can cause conventional RG segmentation method to fail. The proposed algorithm starts merging unallocated neighboring voxels, based on the refined region homogeneity criteria, which is set prior to the segmentation procedure. This procedure continues iteratively until all voxels are assigned with class labels (object or background). Region homogeneity is simply set as the difference between a voxel’s intensity value and the region’s mean.

As a result of our research, we defined two alternative versions of RG segmentation so that users can select the slice-by-slice segmentation in 2D or direct segmentation in 3D, and resultant 3D segmentation can be refined by minimal user interactions. When necessary, a user can reallocate a few seeds to include some pathological parts into the segmented lung regions. This latter step is conducted in 2D, and refined segmentation is immediately included in the final outcome.

The benefit of integrating pathological regions into the lung volume computation is due to the fact that some of those regions may not be included by the RG algorithm because of existence of the pathologies. Furthermore, slice-by-slice IRG is suited best for segmentation of abnormal imaging patterns because the number of slices occupying imaging abnormalities is usually small, and user interaction for this method is not time-consuming. Nevertheless, this part can also be conducted in full 3D version of the region growing algorithm. Additional file 2 shows a demo video of the interactive lung segmentation from a CT scan, in 2D and 3D. Alternatively, one may consider a machine learning-based method to make the whole process fully automated rather than interactive. For example, Sofka’s pathological lung segmentation method [27] may potentially be adapted for small animal CT images. However, it should be noted that the main computational difficulty in fully automated pathological lung segmentation problems is the large anatomical and pathological variability as well as low efficiency due to high computational cost (e.g., Sofka’s proposed method takes an average of 40 to 45 min for a high-resolution CT scan from a human patient).

#### Segmentation of radiotracer activities from PET scans

Segmenting functional regions from PET scans is mandatory in the quantitative evaluation of functional images. It does not only give information about volumetry of functional uptake, but it also provides correct margins for the precise computation of SUV parameters [23,24]. We recently proposed a graph-theoretic segmentation method for radiotracer uptake regions, with ‘non-diffusive appearances’ from PET images, which achieved higher sensitivity and specificity compared to the state-of-the-art methods [16,24]. However, since abnormalities within the lungs of a small animal infectious disease model show ‘diffusive (multi-focal) appearances’ with non-convex geometrical shapes, it may cause graph-based segmentation algorithms to leak into non-object territories within the scene. Therefore, we designed an adaptive thresholding method, suited well specifically for small animal models of pulmonary infections, in addition to the use of our previously proposed graph-based segmentation method (named random walk image segmentation of PET images [28,29]).

Although thresholding-based image segmentation methods are the current convention in clinics, finding globally optimal threshold level is technically challenging. The standard clinical threshold level is 40% to 43% of the PET image histogram in human studies, however, the optimal level can change readily in small animal studies. Therefore, we adapted the conventional FCM segmentation procedure into the regions of the PET image as well as a possibility of getting interactive information from expert radiologists in identifying the near-optimal thresholding level. Once FCM finds the number of participating objects within the scene, our adaptive thresholding method tries to find the near-optimal thresholding level in the vicinity of the object boundaries. The search algorithm in the vicinity of the object boundaries was based on the iterative search algorithm, ultimately finding the maximum entropy value under the histogram of the PET image under consideration. See Additional file 3 for a demo video of the segmentation from a PET scan, in 2D and 3D.

#### Incorporating image registration pipeline for volume change analysis from serial scans

Quantitative measurements of change in radiotracer uptake’s spatial position require serial registration of a longitudinal image sequence from the same subject into a common space. Jointly aligning all image sequences - different scans from the same subject - to a common space allows researchers to visualize how radiotracer uptake are changing over time. This information is critical when identifying disease-related changes, particularly when exploring the severity and extent of the diseases. After aligning them into a common space, segmented structures from the same subject can be compared both locally and globally. However, direct pairwise registration of segmented PET images is not feasible because of limited anatomical markers within the images. Several image registration algorithms have been developed to achieve this goal [24,30-34]. Among them, we used locally affine and globally smooth image registration of serial CT images [24,33] and transfer the resulting deformation fields to the corresponding PET images because the registration method reported in [24,33] has been shown to be much more accurate than simple affine and rigid registrations. With this procedure, one can bring PET images within a common anatomical space (CT).

**Figure 4:**
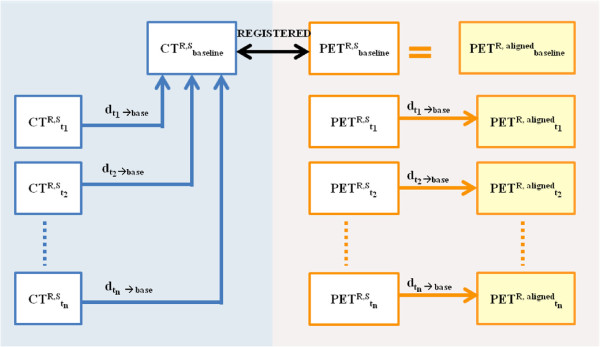
**Scenario of our proposed registration framework.** In the first step (left) for each animal ‘R’, we registered segmented CT volumes (CT ^R,S^) from different time points (*t*_*n*_) to a baseline scan and stored the deformation fields $\left(d_{t_{n}\;\to \;\text{base}}\right)$. Since the PET images are co-registered with these CT scans, we applied the corresponding deformation field to the segmented PET scan (PET ^R,S^) in order to align the PET radiotracer regions over time (PET ^R, aligned^)(right). This process allowed visualization of changes in active PET lesions throughout the imaging study.

The scenario of our proposed registration framework is demonstrated in Figure 4. Our approach includes two different phases. In the first phase (phase I), we aligned all image sequences of CT scans into a baseline scan and stored the deformation fields resulting from registration process. Note that since we already segmented the lungs from CT scans, we performed nonlinear registration using binary lung volumes instead of using the CT gray level images. The key idea was to use CT scans for image registration since CT includes more (consistent) information, and determining spatial correspondences between CT scans is relatively easier compared to PET-to-PET registration. Furthermore, by extracting lung regions from CT scans, we maximized the information available for finding correspondences between scans from different time points. In the second phase (phase II), we applied the stored deformation fields (obtained from first phase, *d*_(*t* → base)_) to the corresponding PET images named parallel transportation [32]. Once transformation fields were applied to PET images to align with their corresponding CT scans, we segmented the radiotracer uptake regions (PET ^R,aligned^). This step can be visualized and analyzed without doing any further steps.

## Results

To assess the efficacy and utility of our segmentation methods (in CT and PET images) quantitatively, we used accuracy measurements which are related to how well segmentation results compare with the true delineation of objects. Ground truths of delineation for the objects were obtained by expert radiologists’ annotations. We also considered efficiency measurements for the segmentation evaluation, where efficiency pertains to the practical viability of the method which is determined by the amount of time required for the computations to provide the user the help needed during segmentation.

### Observer agreement study - evaluation on CT scans

**Figure 5:**
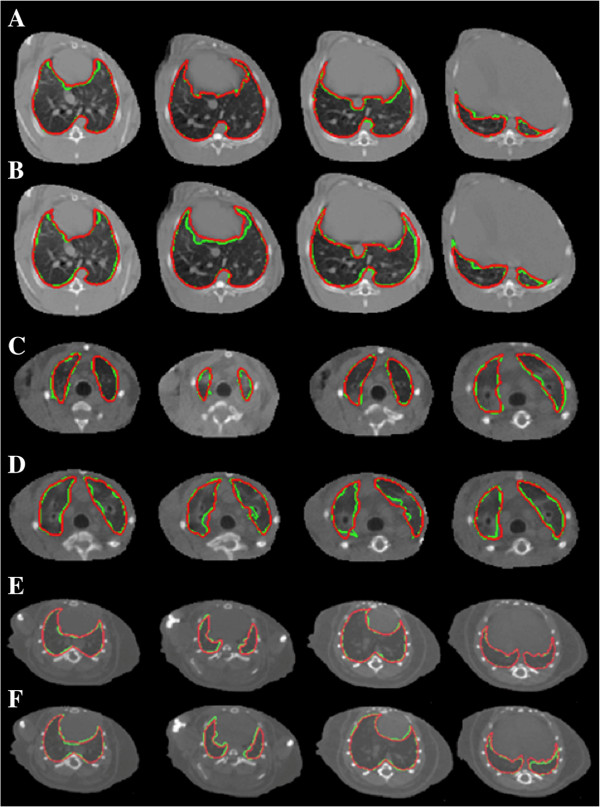
**Intra- and inter-observer variability for different anatomical (CT) slice levels for rabbit, ferret, and mouse animals. (A,C,E)** Multiple CT axial image slices from a rabbit, ferret, and mouse that have been segmented manually using a live-wire algorithm, by the same operator, in two different time points (red, time number 1, green, time number 2). **(B,D,F)** Rabbit and ferret segmentations from two different observers highlighted in red and green, respectively, across multiple lung locations. Intra-observer **(A,C,E)** and inter-observer **(B,D,F)** evaluations of the segmentation process show good agreement across four different lung locations. Quantitative evaluations of these processes are reported in Table 3.

Two expert observers (blinded to the other’s operations) delineated the lungs from CT scans in two different time points. We computed the variance of the entire lung volumes in both healthy and infected animals to determine the intra- and inter-observer agreements by calculating the ratio of tissue samples segmented correctly by the two segmentation methods. The agreements between expert observers were computed over the entire lung volumes of the subjects (namely, overlap ratios of these measurements were computed and averaged). For intra-observer agreement, the rates were computed using the same observer’s segmentation taken days apart. Figure 5 shows intra- and inter-observer variability for different anatomical (CT) slice levels for rabbit, ferret, and mouse animals. The average inter-observer agreement rates were 80.6*%*±9.5*%* in the rabbit, 77.2*%*±11.2*%* in the ferret, and 89.5*%*±3.7*%* in the mouse models, while the intra-observer agreement was 84.7*%*±6.0*%*, 80.3*%*±4.4*%*, and 94.7*%*±1.3*%*, respectively.

**Table 3:** Evaluation of inter/intra-observer agreements over CT and PET image segmentations is shown

**Animals**	**Observer**	**Observer**	**Evaluation of PET**	**Evaluation of**
	**agreement rates**	**agreement rates**	**segmentation between**	**interactive**
	**for CT volume**	**for PET volume**	**consecutive threshold**	**segmentation for**
			**levels (difference by 5%)**	**pathological lung**
Rabbit	Intra(%) 84.7±6.0	Intra (%) 92.3±14.4	DSC (%) 86.0±3.6	DSC(%) 86.0±7.1
	Inter (%) 80.6±9.5	Inter (%) 89.8±14.7	HD (mm) 13.2±2.3	HD (mm) 14.2±2.1
Ferret	Intra(%) 80.3±4.4	Intra(%) 92.7±12.6	DSC(%) 84.0±5.8	DSC(%) 83.4±8.6
	Inter (%) 77.2±11.2	Inter (%) 81.5±16.2	HD (mm) 10.6±1.6	HD (mm) 4.0±1.4
Mouse	Intra (%) 94.7±1.3	Intra (%) 86.6±2.4	DSC(%) 86.5±1.1	DSC(%) 95.1±2.8
	Inter (%) 89.5±3.7	Inter (%) 83.2±3.6	HD (mm) 10.8±1.8	HD (mm) 0.26±0.48

DSC and HD measurements are also reported for evaluating the interactive and adaptive thresholding-based segmentation algorithms compared to ground truths.

### Observer agreement study - evaluation on PET scans

**Figure 6:**
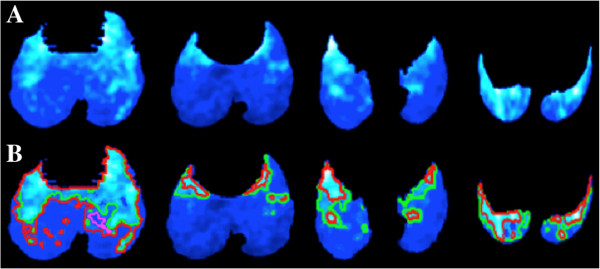
**Segmentation variations by *5% *difference in threshold values. (A)** Multiple PET axial image slices from a rabbit with an active lung infection are shown for demonstration purpose (color code: cold). Areas of light color indicate higher PET intensities, while areas of darker color signify background, noise, or uptake belonging to minimum metabolism. **(B)** Two locally adaptive threshold levels outline areas of interesting uptake regions. Here, only a 5% difference in thresholding level is shown by green and red (i.e., pink outline is the inner boundaries of red outline), respectively.

We compared the functional volume - determined by adaptive thresholding levels - between two expert radiologists who were blinded to their evaluations. Similar to the inter- and intra-observer agreement evaluation in CT segmentation, we computed the variance between the entire lung functional volume in both healthy and pathological lungs in order to determine inter- and intra-observer agreements. Figure 6 shows segmentation variations by setting 5% difference in threshold values. This difference could readily be observed in decisions of the same observer in different time points, or, in decisions of different observers. For quantification of the segmentation process for inter- and intra-observer agreements, we followed the same overlap ratio computation explained in the previous subsection. The mean agreements in PET uptake volume between observers were 89.8*%*±14.7*%* in the rabbit, 81.5*%*±16.2*%* in the ferret, and 83.2*%*±3.6*%* in the mouse models, while the mean intra-observer agreements were 92.3*%*±14.4*%*, 92.7*%*±12.6*%*, and 86.6*%*±2.4*%*, respectively. These intra-observer and inter-observer agreements are good despite the difficulties in determining global thresholding levels.

### Evaluation of the interactive segmentation method on CT scans

**Figure 7:**
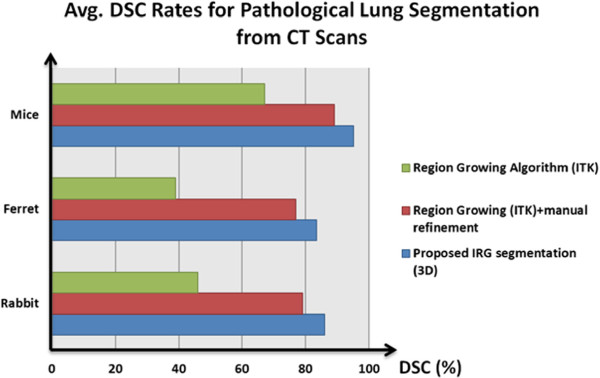
**Average DSC rates for pathological lung segmentation from CT scans.** Average DSC rates for pathological lung segmentation results are given in comparison with frequently used region growing algorithm (from ITK repository), with and without using manual refinements.

**Figure 8:**
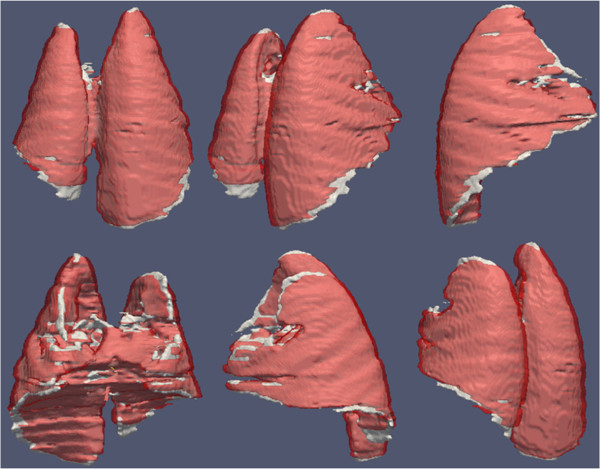
**Performance of the IRG segmentation method compared to ground truth delineation.** Segmented lung volume by expert (red) and interactive region growing algorithm (white) were overlaid for qualitative assessment. High volume overlaps and small deviation of the boundary differences can easily be observed from these images. Columns and rows show the different views of the same animal’s (rabbit) segmented lung volumes.

We evaluated our proposed IRG segmentation method and summarized our results in Table 3. While obtaining the results, we averaged the rates obtained from ground truths in order to avoid any bias towards a particular observer’s evaluation, as complied with the standard evaluation techniques [19,20]. We tested segmentation accuracy and efficiency in both normal and pathological lungs and observed a DSC of 93.4*%*±4.5*%* and 86.0*%*±7.1*%*, respectively. The mean HD between different segmentation was 4.0±1.4 mm in subjects with normal lungs and 4.5*%*±1.4 mm in subjects with pathological abnormalities. Furthermore, Pearson correlation between estimated anatomical volume of lesions by the proposed method and the ground truth was *R*^2^=0.8922,*p*<0.01. We also compared the segmentation results to the ground truth delineations as well as the commonly used RG algorithm from ITK implementation [35]. Figure 7 summarizes the average DSC rates for pathological lung segmentation for all small animal models in comparison with conventional RG method (ITK implementation), with and without using interactive refinements. Note that the proposed algorithm has higher rates than the conventional RG algorithm but has similar performances if the user interaction is allowed to refine the FP regions. Moreover, we qualitatively show the performance of the IRG segmentation method (shown in white) compared to ground truth delineation (shown in red) in Figure 8. Columns and rows of the figure show different views of the same lung segmented from a CT scan of a rabbit. Note that high volume overlap ratios are observed.

### Evaluation of the adaptive thresholding method on PET scans

We evaluated the utility of our adaptive thresholding method for functional volumes in the small animal model by comparing the areas delineated by different threshold levels. Our observations show that clinical convention is usually suited for the segmentation experiments of PET scans for non-infected small animals, which is to set 40% of the overall histogram as a threshold value. This situation might change when infection exists: 60% to 65% of the overall histogram was usually set as a threshold value in later weeks. Moreover, we also observed that expert observers agreed within 5% of this threshold value. Therefore, starting at 20% of the PET ^S^ image histogram and ending at 90% as threshold values, we computed the DSC and HD between threshold levels varying by 5% of the image’s histogram (i.e., 20% and 25%, 25% and 30%, etc.) and then averaged DSC and HD values over different threshold values and different subjects. The DSC and HD for rabbit, ferret, and mouse are reported in Table 3. Similar to the evaluation of segmentation results from CT images in the previous subsection, we averaged the rates obtained from ground truths in order to avoid any bias towards a particular observer’s evaluation as complied with the standard evaluation techniques [19,20]. Furthermore, Pearson correlation between estimated metabolic volume of lesions by the proposed method and the ground truth was found to be *R*^2^=0.8664,*p*<0.01.

**Figure 9:**
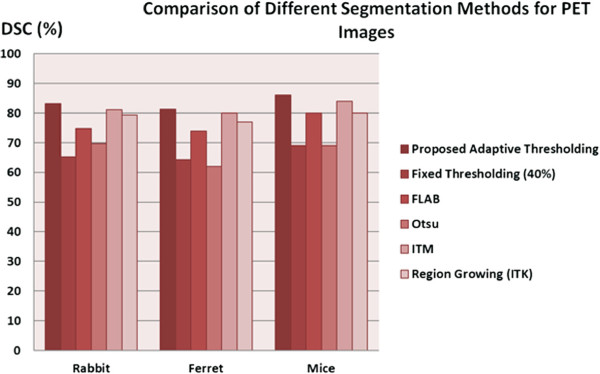
**Comparison of proposed segmentation method with the state-of-the-art image segmentation methods.** The state-of-the-art image segmentation methods for PET scans from small animals are compared to our proposed segmentation method.

**Figure 10:**
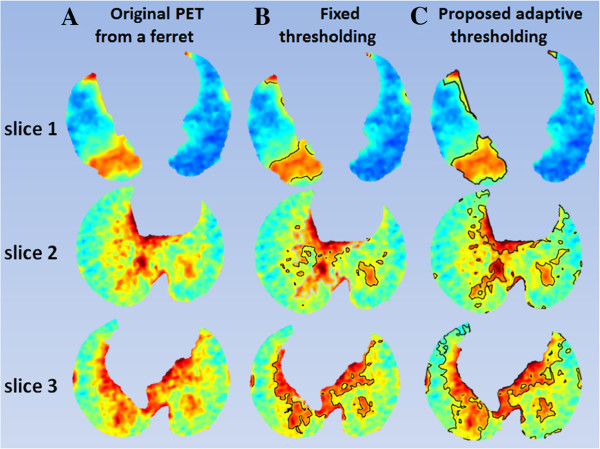
**Three different PET slices of a ferret are shown in the first column.** While PET slices in column **(A)** show original images, delineations resulting from the fixed and the proposed adaptive thresholding methods are shown in columns **(B)** and **(C)**, respectively.

We compared our proposed adaptive thresholding method with the state-of-the-art PET image segmentation methods, including RG [35], iterative thresholding (ITM) [36], adaptive Otsu thresholding [37], and fuzzy locally adaptive Bayesian method (FLAB) [38]. Figure 9 shows the average DSC rates for this comparison for all small animal models used in our experiments. Note that our proposed algorithm was superior to the state-of-the-art methods among which the performance of the ITM and region growing methods were close to each other. Furthermore, we observed that FLAB was superior to Otsu and fixed thresholding when uptake regions were more homogeneous. In addition to the quantitative results and comparison given above, we also demonstrated qualitatively our proposed adaptive thresholding method in comparison with a fixed thresholding method for a given ferret PET image. As exampled in Figure 10, three PET slices were taken from different anatomical locations of the selected ferret in each row, and the resulting fixed and proposed adaptive thresholding based segmentation are given in the second (Figure 10B) and third (Figure 10C) columns, respectively. Since the fixed thresholding method did not take into account the intensity similarities, as opposed to FCM, the resulting delineations (Figure 10B) were sub-optimal. On the other hand, the proposed adaptive thresholding method used FCM (Figure 10C) to refine the segmentation boundary based on the initial delineations, therefore, the refined delineations were visually plausible as complementary to the quantitative results given in Table 3 and Figure 9.

### Evaluation of image registration

In order to quantitatively assess the registration accuracy, anatomical landmark points were manually placed in a subset of selected small animals’ images on anatomically distinct regions or points by an expert radiologist [39]. Similarly, the corresponding landmarks were manually identified in the follow-up scans. This process was repeated for small animal images of the all types. We used a total of 120, 90, and 60 landmark points for rabbits, ferrets, and mice images, respectively. Landmarks were grouped into head, lung, liver, and skin body equally when manually identified by the experts. Note that a grid of 15 landmark points per organ was often shown to be sufficient enough for landmark-based registration and evaluation for small animal images in the literature [40]. The minimum (min), mean, maximum (max), and standard deviation (std) of the landmark distances between baseline and follow-up scans after deformable registration were recorded. Table 4 shows the average pairwise image registration error (i.e., landmark distance) for the whole experiment. The landmark-based registration errors show that the proposed registration framework - using parallel transportation of deformation fields from CT-to-CT registration into PET-to-PET registration - had high registration accuracy as shown by the small landmark distances. We also conducted a *t* test between registration errors resulted from different animals, and we observed that there was no statistically significant difference (*p*>0.05) between registration results and different images of the small animals.

**Table 4:** Statistics of landmarks error (in mm) using deformable group-wise registration of segmented CT images

**Landmark distance**	**Min (mm)**	**Mean (mm)**	**Max (mm)**	**Std (mm)**
Rabbit	1.01	2.66	4.50	1.74
Ferret	1.86	3.93	4.65	1.24
Mouse	0.23	2.52	6.00	1.62

**Figure 11:**
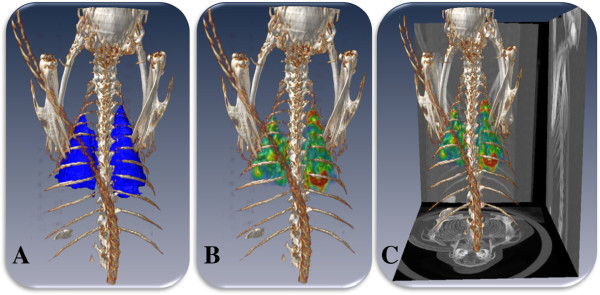
**Combined hybrid visualizations of qualitative and quantitative evaluations in a single scene.** Qualitative (volume rendering) and quantitative (surface rendering) evaluations of the segmented **(A)** lung and **(B)** PET uptake pertaining to the lungs are shown. In **(C)**, rendered body region and PET uptake are overlaid with CT projection views on the sides in order to further simplify anatomical localization of the anatomical and functional information (rabbit number 3 at week 5).

**Figure 12:**
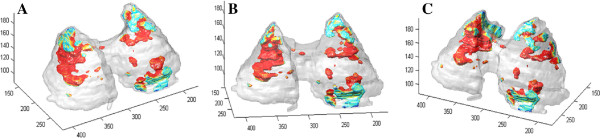
**Simplified quantitative visualization in three different views of a segmented lung surface.** Simplified view for a segmented lung surface from a CT scan of a rabbit. **(A,B,C)** Segmented significant uptake regions from PET images are shown in three different positions. While red indicates the highest uptake, green indicates the relatively lower uptake but still within the significant uptake regions delineated from PET images.

Figure 11 gives combined hybrid visualizations of qualitative and quantitative evaluations in a single scene. In Figure 11A, the volume-rendered body region of a rabbit is combined with quantitative surface information of the segmented lungs, while Figure 11B shows the PET image volume pertaining to the lungs in color code after the morphological operations (i.e., PET ^M^) within the same volume-rendered body region of the rabbit. Additionally, Figure 11C demonstrates the projection-based view from the CT scans to further enhance anatomical localization of structures along with functional lung information. Figure 12 shows a simplified quantitative visualization in three different views of a segmented lung surface from a CT scan of a rabbit overlaid onto the segmented significant uptake regions from PET images. Note that in this quantitative visualization, objects are rendered in surface levels after segmentation process. Color code is used to indicate the hottest uptake regions within the significant uptake regions delineated from PET images.

### Computational cost, parameter training, and efficiency

All programs used in this study were developed using C++ gcc 4.5 (copyright 2010 Free Software Foundation) on a Linux platform (Ubuntu, Canonical USA Inc., Lexington, MA, USA), and all statistical computations and user interface to access segmentation, registration, and quantification algorithms were processed in Matlab (copyright 2010 MathWorks Inc., Natick, MA, USA). All the programs were executed on an Intel ^*Ⓡ*^ Core(TM) i7 CPU 930 at 2.80 GHz with 12 GB RAM workstation. While manual segmentation of the lung regions from CT scans takes around 60 to 90 min depending on the severity of the disease, the interactive lung segmentation takes only 16.52 s on average for approximately 250 slices. This timing can take up to 10 min for very severe cases, where a refinement step with interactive one-click process for some slices is necessary. On the other hand, segmentation of PET images takes only 11 s on average for approximately 250 slices.

Users deem it necessary to incorporate some pre-processing steps prior to actual segmentation of CT images or SUV analysis of PET images. Our proposed computational framework offers this flexibility of pre-processing images prior to conducting image analysis, as exemplified partially in the Additional files 1 and 2 demo videos. There may be other necessary steps for proper segmentation due to unconventional image features or scanner dependent variations. For instance, a scan may be loaded into the system in a prone position rather than supine, and PET correspondence of the anatomical image may be aligned in opposite direction (head to toe alignment), considering all these issues in a fully automated way requires an extensive search and individualized event detection algorithms. It may take longer for a fully automated computer algorithm to identify these problems first and take the appropriate actions due to the difficulty of ‘recognizing’ an unexpected problem. On the other hand, a human observer can easily handle these issues by visual inspection and take the necessary action within seconds. This is one of the main reasons that many research tools, including our proposed computational framework, are being developed in a way to allow users to efficiently interact with the tools [41].

### Imaging findings and longitudinal analysis of pulmonary infections

One of the clinical research questions for this study was to investigate the immune response in the lungs to pulmonary infections over time through FDG PET-CT imaging. To demonstrate how our proposed computational pipeline would be used for longitudinal analysis of pulmonary infection, we present here an illustrative example of the TB rabbit model. For the TB rabbit model, as stated earlier, the rabbits were infected with TB and imaged longitudinally at various time points (0, 5, 10, 15, 20, 30, and 38 weeks) after initial baseline scans. Once the images are acquired, the quantification of inflammatory response can be carried out using the proposed computational framework. Since the amount of change of functional uptake volume and their strength from PET images as well as the change of pathological volume from CT images can be used as reliable markers for showing disease progression in longitudinal manner, we quantified the disease progression and resolution with these three markers. For the amount of functional uptake volume, the volume of the significant uptake regions automatically segmented from the PET images was used. For the delineated region’s signal strength, maximum SUV (i.e., SUV _max_) was used. Lastly, for the pathological volume marker: the volume of segmented pathologies from the CT images was used. In other words, 

•The SUV _max_ was used to show how ‘intense’ the disease is

•The functional uptake volume was used to demonstrate how ‘much’ immune response and inflammation was present, and

•The pathological volume was used to show how much the parenchyma of the lungs had been altered due to the infection as seen on CT.

Furthermore, researchers can easily add other quantitative metrics to our proposed framework, such as SUV _mean_ (the mean SUV over the functional uptake volume) and SUV _lbm_ (lean body mass-based SUV), which can give an even more in-depth look at the disease progression. However, novel exploration of quantitative and semi-quantitative metrics is outside the scope of this paper.

**Figure 13:**
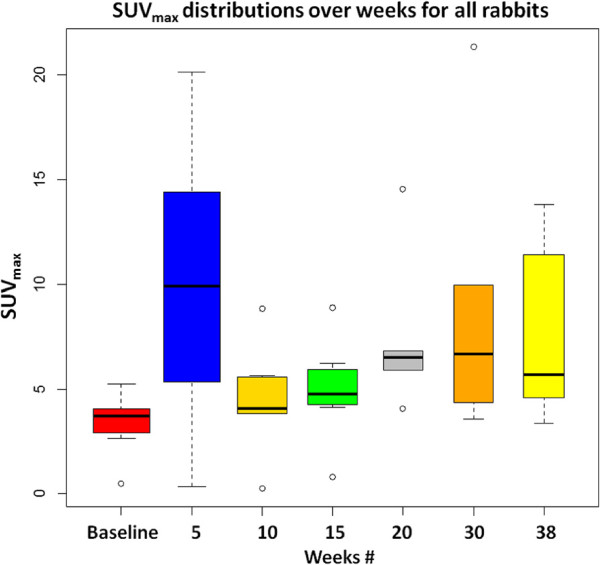
**SUV**_***max ***_**distributions for all rabbits are shown over weeks.**

**Figure 14:**
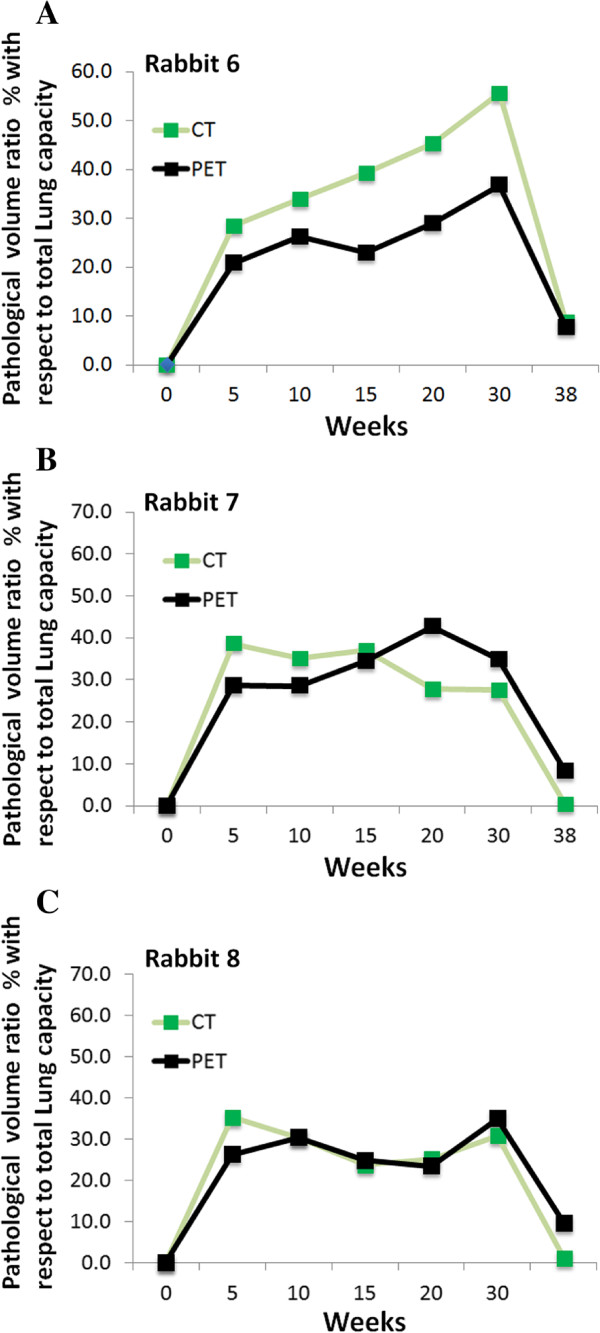
**Metabolic and gross pathology volume percentage of the lungs for three different rabbits.** The pathological lung volume ratio (both functional and structural) with respect to total lung capacity over weeks are plotted for individual rabbits: rabbit 6 **(A)**, rabbit 7 **(B)**, and rabbit 8 **(C)**, respectively.

**Figure 15:**
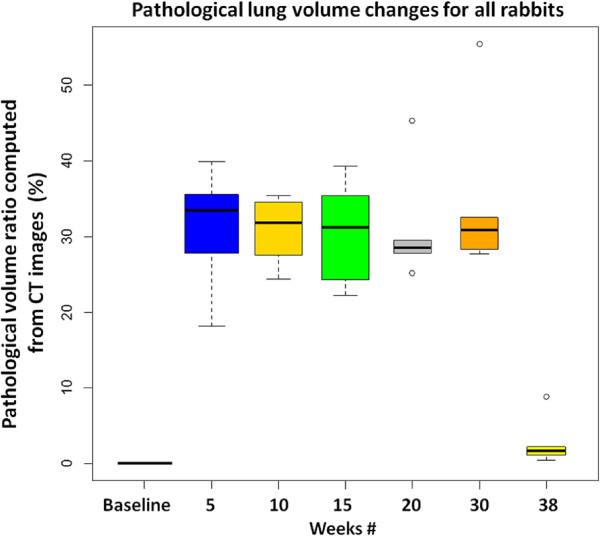
**Pathological lung volume changes for all rabbits.** The pathological lung volume ratios, computed through delineation of CT images, and their longitudinal changes are plotted for all rabbits.

**Figure 16:**
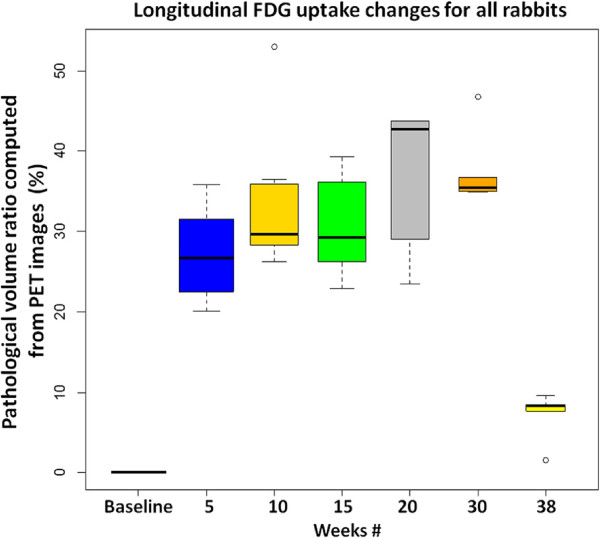
**Longitudinal FDG uptake changes for all rabbits.** Longitudinal FDG uptake volume changes, computed through delineation of PET images, are plotted for all rabbits.

After registration and segmentation, the SUV _max_ of the delineated lesions was calculated by taking the highest SUV within the functional uptake volume. Figure 13 shows the progression of SUV _max_ in the serial scans of all rabbits from 0 to 38 weeks. It was noted that the highest variation in SUV _max_ was observed soon after the animals were infected with TB, and in all weeks, SUV _max_ was higher compared to the metabolic activities measured at baseline scans. It was revealed from the figures that the general trend of the change in SUV _max_ and the functional uptake volume percentage over time was similar. Figure 14a,b,c shows metabolic and gross pathology volume percentage of the lungs for three different rabbits (i.e., rabbit numbers 6, 7, and 8). Figures 15 and 16 summarize the pathological lung percentage and FDG volume uptake changes for all rabbits over all weeks, respectively. It is important to note that even though there is not necessarily a one-to-one correspondence between the functional and structural volume ratio (i.e., not all areas of consolidations have significant FDG uptake), the trends of the longitudinal pathological volume changes in these figures coincide with the experimental evaluations including qualitative visual inspection by the participating radiologists and nuclear physicians.

In order to visualize longitudinal changes of significant uptake within the lung regions, three movie samples representing the longitudinal FDG uptake of rabbits numbers 6 and 7 and ferret number 2,214 are provided in Additional files 4, 5, and 6. For these visualizations, the registration pipeline was the most crucial part where the deformation fields from CT-to-CT registration were obtained and transported into the PET image correspondences in order to overlay segmented functional uptake of the same animal’s different scans within the same spatial domain. This process enhances the understanding of how disease is progressing over time as well as indicating the spatial localization of pathology, which aids in taking corresponding histology slices for biopsy purposes. Note also that our visualizations are based on surface renderings of the segmented objects, therefore, quantitative surface and volume information are available in contrast to commonly used qualitative volume rendering. In the demo movie of Additional file 2, we kept the lung and trachea together for visualization purpose only (i.e., to guide pathologists to identify the anatomical slice level when doing histology analysis), however, for quantitative evaluation of the lung volume and related calculations (as demonstrated in the movies of Additional files 4, 5, and 6), we used the conventional trachea removal tool, which was based on a simple Hough transform. Relevant information regarding this conventional method can be found in [42,43].

## Discussion

Our study presents a computational platform for automated and interactive methods for measuring areas of abnormal uptake on PET and corresponding anatomical structures on CT, with longitudinal assessment of lesion volumes, in order to increase the efficiency and quality of quantitative imaging studies. Although some of the methodologies presented in this manuscript such as segmentation from CT scans, PET segmentation, and co-registration aspects of the image processing tasks have been established more or less in the literature, a computational pipeline that takes into account the unique challenges of PET-CT imaging, particularly for small animal models and pulmonary infections, has not previously been explored. Considering (a) the exploration of the feasibility of PET-CT multi-modality imaging in small animals and (b) the unique challenges of pulmonary infections (i.e., multi-focal uptake in PET, diffusive abnormal imaging patterns in CT, the challenge of segmenting pathological lung regions, etc.), to the best of our knowledge, our study is the first one taking into account all these difficulties and propose a reliable, fast, and accurate computational platform to be used in pre-clinical and clinical studies.

For this study, the FDG radiotracer was used because it is the most commonly used agent for molecular imaging, but the method can also be applied to any PET tracer. In addition to the CT anatomical imaging, our proposed computational framework can also be used with MRI data fused with PET imaging, therefore, multi-modal anatomical images with functional correspondences can readily be quantified within our system. Moreover, the study presented herein focuses on small animals - rabbits, ferrets, and mice. Given the need for increasing the efficiency, speed, and quality of molecular imaging in pre-clinical research, this application of automated quantitative imaging studies for small animal models is warranted.

Our study applies to infectious disease in particular because this disease category has some unique challenges such as multi-focal heterogeneous lesions with rapid progression, necessitating more efficient methods for quantifying molecular imaging data. Given the accuracy and efficiency of our presented results, this methodology can also be applied to measuring tumor volumes in oncologic research, because diffuse PET uptake patterns and intensities caused by inflammation and infections can have very similar characteristics compared to those caused by cancer within the thoracic cavity [1]. Indeed, the segmentation of diffuse patterns (both from CT and PET) is much more challenging compared to the segmentation of solid tumors, hence, our methods can readily be used within different frameworks concerning cancer-related problems. Our computational platform can also provide automated tumor volume measurement using the same approach as the demonstrated measurement of abnormal PET-CT findings in infection to provide serial volumetric data and longitudinal changes in SUV intensity.

In this paper, infectious disease progression was analyzed and reported for small animals, however, the ultimate aim within the scope of this research is to find and understand the progression of infectious diseases in human subjects as well. To the best of our knowledge, there is no systematic study exploring this longitudinal phenomena in human clinical trials yet. Based on our previous research [44-46] and some other works showing the imaging findings of pulmonary infections, there are certain similarities in image analysis of pre-clinical and clinical subjects. Consolidations and GGOs are the two main imaging patterns from CT images which are observed both in human subjects and small animals. Similarly, SUV analysis of lesions detected from PET images is conducted in the same way for human subjects and small animals [14]. On the other hand, we can argue that the spectrum of the observed imaging patterns from CT images will be wider than the observed imaging patterns of small animals. For instance, in parainfluenza and H1N1 infections in human subjects [44-46], we observed not only GGO and consolidations but also tree-in-bud, nodules, pleural effusions, and interstitial thickening. Therefore, additional individual analysis of how those patterns change longitudinally (i.e., shape and volume) could bring certain additional insights into the exploration of pulmonary infection dynamics in human subjects. However, these analyses require separate CAD systems for each abnormal imaging pattern pertaining to pulmonary infections.

Future studies will aim to correlate the automated algorithm’s performance with other parameters of respiratory disease such as histopathology, inflammatory cell counts, and viral/bacterial titers. For this purpose, we are currently developing novel methods for automatic airway extraction and quantification as well as simultaneous co-segmentation and co-evaluation of CT, MRI, and PET multi-modality images [28,47].

## Conclusions

Experimental results showed that automated computer-detection of lesions in pulmonary infections through our proposed computational pipeline is efficient and accurate relative to the manual approaches and thereby increases reproducibility of automated quantification of disease immunopathology in small animal models. The immense importance of infectious disease imaging in small animal models is based on the fundamental necessity to study disease pathogenesis and host immune response as well as to assess therapeutic measures such as medications and vaccines, on a time scale basis on the same subject(s). Measuring disease follow-up is especially important because the evaluation of the natural progression of untreated disease as compared to the regression of treated disease requires accurate serial quantification over time.

Our proposed computational platform is novel and unique, which is suited well for analysis and quantification of pulmonary infections in pre-clinical and clinical research questions. We demonstrated our computational framework’s feasibility and robustness using longitudinal PET and CT images obtained from multiple small animals and compared its performance to expert delineations.We concluded from the clinical findings derived from the proposed computational pipeline that serial PET-CT imaging is an invaluable hybrid modality for tracking pulmonary infections in small animals and has great potential to become the state-of-the-art imaging tool in routine clinics for pulmonary infections. In a broader perspective, we hope that the methods and experiments presented here will support further exploration of infectious diseases in different small animal models.

## Abbreviations

CT: Computed tomography; DSC: Dice similarity coefficient; FCM: Fuzzy c-means; FDG: Fluorodeoxyglucose; FLAB: Fuzzy locally adaptive Bayesian; FP: False positive; H1N1: Swine flu; HD: Haussdorf distance; IRG: Interactive region growing; ITM: Iterative thresholding method; MRI: Magnetic resonance imaging; PET: Positron emission tomography; RG: Region growing; STD: Standard deviation; SUV: Standardized uptake value; TB: Tuberculosis.

## Competing interests

The authors declare no competing financial interests.

## Authors’ contributions

DJM, UB, SJ, and CBJ co-initiated the project and designed the research. BL, BD, WRB, and SJ designed and performed the lab experiments including imaging of rabbits and mice, while CBJ designed and performed the lab experiments for ferret data. UB and KMJ developed the image processing algorithms for quantitative and longitudinal analysis of small animal images. UB wrote the software for all algorithms. UB, BF, and KMJ prepared all figures and tables. DJM and UB designed a segmentation evaluation test for inter- and intra-operator variation derivation. UB and KMJ conducted all statistical tests. Supplementary videos and materials were prepared by UB, BF, and DJM. The manuscript was written by UB, KMJ, and DJM, and was critically revised by SJ, CBJ, BL, WRB, BF, and BD. All authors read and approved the final version of the manuscript.

## Supplementary Material

Additional file 1 Supplementary information for image registration, image segmentation, and formulation for DSC and HD evaluation is given. Click here for file

Additional file 2 **Movie 1.** Segmentation and quantification of lungs from CT. Please click the link below (using google account may avoid possible browser problems): https://docs.google.com/file/d/0B2XUKmP9htKTNDM5ZmtOY2Q2SUk/edit?usp=sharing. Click here for file

Additional file 3 **Movie 2.** Segmentation and quantification of PET images. Please click the link below (using google account may avoid possible browser problems): https://docs.google.com/file/d/0B2XUKmP9htKTQ2xoM01JWDZSUUU/edit?usp=sharing. Click here for file

Additional file 4 **Movie 3.** Visualization of FDG uptake longitudinally (rabbit 6). Please click the link below (using google account may avoid possible browser problems): https://docs.google.com/file/d/0B2XUKmP9htKTVTI4bFZ6NTJWTlk/edit?pli=1. Click here for file

Additional file 5 **Movie 4.** Visualization of FDG uptake longitudinally (rabbit 7). Please click the link below (using google account may avoid possible browser problems): https://docs.google.com/file/d/0B2XUKmP9htKTcE5EMUltbUpJd0E/edit?usp=sharing. Click here for file

Additional file 6 **Movie 5.** Visualization of FDG uptake longitudinally (ferret 2214). Please click the link below (using google account may avoid possible browser problems): https://docs.google.com/file/d/0B2XUKmP9htKTaUkyQzVzbDZaenc/edit?usp=sharing. Click here for file
