# Supplementary material for: A computational pipeline for quantification of pulmonary infections in small animal models using serial PET-CT imaging
Source: EJNMMI Res. 2013 Jul 23;3:55. doi: 10.1186/2191-219X-3-55 (PMC3734217; doi:10.1186/2191-219X-3-55)
Supplement: Additional file 1 — Supplementary information for image registration, image segmentation, and formulation for DSC and HD evaluation is given. [file 2191-219X-3-55-S1.docx]

SUPPLEMENTARY INFORMATION

**A Computational Pipeline for Quantification of Pulmonary Infections in Small Animal Models using Serial PET-CT Imaging**

## Supplement 1

### Image Registration

Image registration involves the development of a reasonable transformation between a pair of images and the source and target, such that the similarity between the transformed source image (registered source) and target image is optimum. The similarity measure should capture both large and small-scale deformations. Based on the nature of the transformation, registration types can be classified as rigid, affine, and deformable (or elastic). In our proposed computational platform, we use affine registration to register CT and PET volumes to get rid of positional, scale, and rotational differences between functional and anatomical images. In order to handle local differences between two images and analyze them longitudinally, a deformable registration is necessary. For this purpose, we use group-wise deformable image registration in longitudinal images.

For the affine image registration method, we follow the approach reported in [1], where a 4x4 affine transformation matrix *A* was estimated between PET and CT images in 3D. A simple way to estimate *A* was to minimize quadratic error function *E(A)* defined as

$$E(A)\approx\sum_{v\in\text{ Overall Image Domain}} [S(v)-T(A.v)]^{2}$$

where *v* denotes spatial coordinates, S and T show source and target images (i.e., PET and CT), respectively. In addition to this function, an additional constraint -smoothness constraint (*E_smooth_*)- was added to the quadratic error function minimization to avoid the optimization problem of getting stuck in the local optima and provide smooth deformations between images. Total quadratic function (*E(A)*+*E_smooth_*) can be minimized by differentiating it, with respect to unknown affine transformation matrix A and equating it to zero. For derivative of smoothness term (*E_smooth_*), one may use different smoothing approaches to penalize very large deformations and keep the transformation smooth. Herein, we use the magnitude of the gradient of the flow between images as a penalize function. **A multi-level pyramid with four levels was used for affine registration. In each level of the pyramid, a single global affine transform was estimated using the minimization of quadratic error function defined above. At most 40 iterations were allowed for the affine registration in each level. Temporal and spatial derivatives of the images were computed using 2-tap and 3-tap filters, respectively. Other affine registration methods could also be used for this purpose; however, with the current method, (i) there were fewer parameters to be set prior to registration, (ii) possible intensity variations between images (if any) were handled automatically, and (iii) the fast convergence was assured. Further technical details on the globally smooth affine registration can be found in [1]. A sample code can be found in [2].**

For longitudinal quantification of PET uptakes for a particular subject, we used deformable image registration using intensity based similarity measures and B-spline parametric deformation models, as indicated in [7, 9]. Given that *p* denotes probabilities of a set of voxels *s*, *R* denotes the subject, *S* indicates that segmentation process has been done, and *t* shows the particular time point, then the registration algorithm basically estimates a transformation function, *d*, using a similarity distance measure *D* (which is normalized mutual information (NMI) in our case), and a constant α as follows:

$$d_{t\to base}=argmin_{\mu} (-D\left[\mu;CT^{R,S}_{t},CT^{R,S}_{baseline}\right]+\alpha M(\mu)),$$

where NMI between two image being registered is defined as

$$D\left[\mu;CT^{R,S}_{t},CT^{R,S}_{baseline}\right]=NMI\left[\mu;CT^{R,S}_{t},CT^{R,S}_{baseline}\right]=\frac{H(\mu;CT^{R,S}_{t})+H(\mu;CT^{R,S}_{baseline})}{H(\mu;CT^{R,S}_{t},CT^{R,S}_{baseline})}.$$

*H* is widely used measure of information called entropy and computed as $H(\mu;.)=-\sum_{s} p\left(\mu;s\right)\log p(\mu;s)$.

Note that $p(\mu;s)$is marginal discrete probabilities of the image, *M* is a regularization term used to penalize second order derivatives of the deformation field *d* in order to have smooth transitions between images, and $\mu$ is parameter vector containing the deformation field coefficient that we seek to find (i.e., $d_{t\to base}$). More details about NMI based deformable registration process can be found in [3-7, 9].

**Deformable registration of two CT scans was performed first by using an affine registration with 12 parameters (3 for translation, 3 for rotation, 3 for anisotropic scaling, and 3 for shears) with 20K iteration at most followed by a free form deformation (FFD) method [9]. To expedite the registration process, we used nearly 700K spatial samples from source and target CT images to compute NMI over the pairwise mutual information histogram with 500 bins. In total, depending on the misalignment of the images, the registration of the longitudinal CT images took 5-10 minutes of CPU time on an Intel (R) Core(TM) i7 CPU 930 at 2.80 GHz, with a 12 GB RAM workstation. Details of the FFD based registration method, its validation, and evaluation can be found in [9]. A free FFD implementation (ITK) can be found in [10].**

### Image Segmentation

*Segmentation of PET Images:* Unlike classical thresholding algorithms, we force our segmentations to be smooth. Therefore, we compute the histogram of the image first and then smooth it via a Gaussian smoothing operation. Next, we find the most significant peaks in the histogram, depending on the number of tissue classes obtained by the user (i.e., adaptation from fuzzy c-means (FCM) algorithm [8]). Finally, the user integrated threshold value based segmentation and the FCM based segmentation are compared by a fitting function which simply evaluates the difference of average intensity values from segmentations, by thresholding and FCM based methods and by updating thresholding values that average intensity values belonging to final segmentation; it should be equal to the average of the intensity values of objects obtained by FCM and thresholding based segmentations.

*Segmentation of CT Images:* Interactive region growing algorithm (IRG) was proposed to use in order to segment the lungs from CT images. Voxel intensities -within the neighborhood of the seed- provided by the expert/user, are iteratively examined, based on the homogeneity criteria and the examined voxels’ intensity values, when boundaries of the objects are found. In our proposed framework, users have the opportunity to replace more seeds or define background regions if necessary (i.e., pathological regions may not be covered in the initial segmentation). For the whole segmentation, naïve region growing segmentation and additional segmentation results, produced from the users defined seeds interactively, are combined to serve as final segmentation. Prior to final segmentation, one may incorporate a simple fill-region and smoothing operator to have connected regions in lung segmentation with a smooth boundary. A brief overview of the region growing technical aspects can be found in [8].

### Formulation for DSC and HD Evaluation

Apart from providing qualitative framework to clinicians for their visual assessment on segmentations (of CT and PET images), quantification of the segmentations are usually required and assessed by dice similarity coefficient (DSC) and Hausdorff distance (HD). In our segmentation experiments -both for functional and anatomical data- high DSC and low HD values were observed, meaning that highly accurate segmentations were obtained. A brief description of these segmentation evaluation metrics is below.

DSC measures the agreement of segmentation with ground truth segmentation. Assuming segmented volume (or set) is denoted by *V_1_* and the ground truth segmentation is shown by *V_2_.* Then, DSC is simply an overlap ratio of these two volumes and computed as

$$DSC\left(V_{1},V_{2}\right)=100\frac{2.|V_{1}\cap V_{2}|}{|V_{1}|+|V_{2}|}.$$

More precisely, as indicated in supplementary Figure 1 below, overlap of two segmentations ($V_{1}\cap V_{2}$) indicates true positive volume fraction (TP) of the segmentation performance. While calculating the overall performance, both false positive (FP) and false negative (FN) fractions are also considered in DSC computation to have the correct segmentation accuracy. Moreover, DSC can also be written in the form of sensitivity and specificity parameters as

$$DSC\left(V_{1},V_{2}\right)=100\frac{2.TP}{\left(FP+TP\right)+(TP+FN)}.$$

A value of DSC=100 indicates a perfect agreement, while a value of 0 indicates no overlap observed.


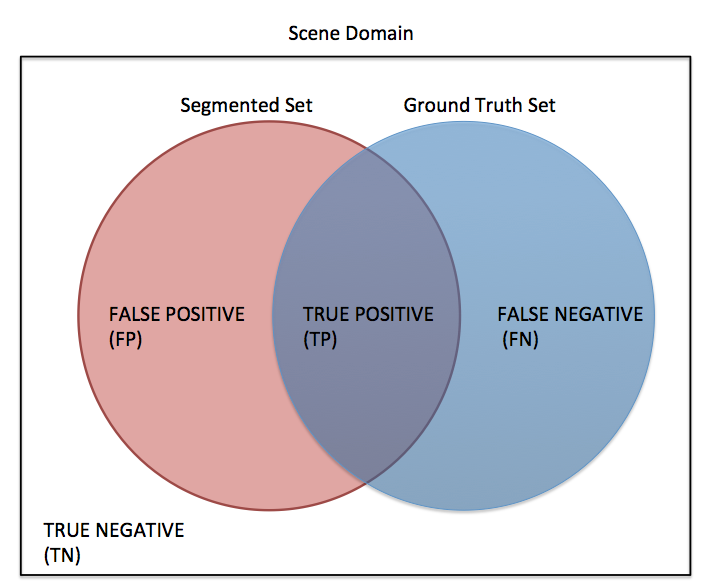


**Figure 1:** Schematic representation of segmentation evaluation framework.

While DSC is a measure of area/volume agreement, HD, on the other hand, is a metric measuring how two segmented objects’ boundaries are in agreement. In other words, how close/far the segmented boundaries are from each other. HD is defined as

$$HD\left(X,Y\right)=\max\left\{sup_{x\in X}inf_{y\in Y} d\left(x,y\right), sup_{y\in Y}inf_{x\in X} d\left(x,y\right)\right\},$$

where *d* is Euclidean distance between points x and y, sup and inf represent supremum and infimum, and X and Y indicate boundaries of segmented object and ground truth computed from boundary operation over volumes (i.e., $HD\left(X,Y\right)=HD\left(\partial V_{1}=X,\partial V_{2}=Y\right), \partial$represents boundary). Visual assessment of HD computation is sketched in supplementary Figure 2 below:


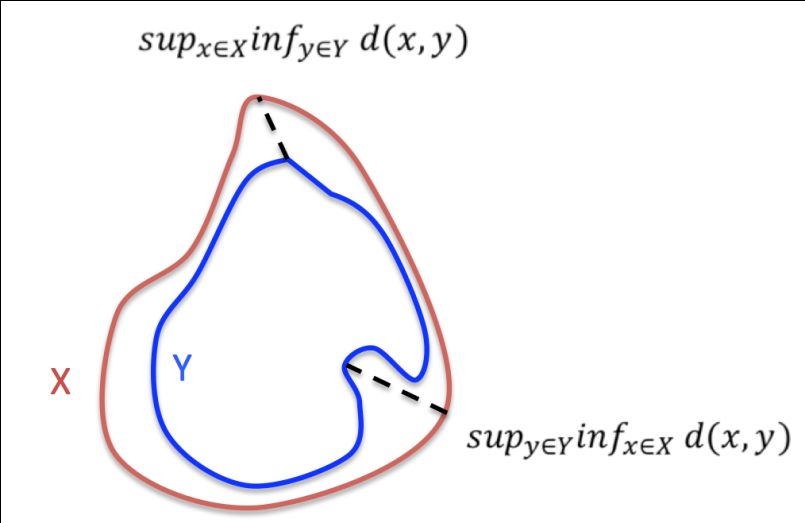


**Figure 2:** Schematic illustration of Hausdorff computation. X (segmented object’s boundary) and Y (boundary of ground truth segmentation) are used to find the largest disagreement between boundaries. Maximum of the found distances from set X to Y and from Y to X are set as a HD measurement between the boundaries.

In its simplest definition, HD can be regarded as a metric showing the farthest distance between boundaries of two segmentations, thus, indicating boundary agreement. As it is schematically demonstrated, two segmentations can have moderately high DSC values due to large overlap; however, boundaries may not agree well. Therefore, DSC agreements -together with HD measurements- give more and accurate quantification information about segmentation results, as we reported both evaluation metrics in the manuscript to give a complete evaluation of segmentation accuracies in a standard scale.

**REFERENCES FOR SUPPLEMENT 1**

1. Periaswamy, S., Farid, H. Elastic registration in the presence of intensity variations. *IEEE Transactions on Medical Imaging* 22(7), 865-874 (2003).
2. Locally Affine Globally Smooth Image Registration Matlab source code: <http://www.cs.dartmouth.edu/farid/Hany_Farid/Research/Entries/2011/5/17_Image_Registration.html> (Last checked: 22 May 2012).
3. Xue, Z., Shen, D.G., Davatzikos, C. CLASSIC: Consistent longitudinal alignment and segmentation for serial image computing. *Neuroimage* 30(2), 388-99 (2006).
4. Liao, S., Jia, H.J., Wu, G.R., Shen, D.G., Neuroimaging, A.D. A novel framework for longitudinal atlas construction with groupwise registration of subject image sequences. *Neuroimage* 59(2), 1275-89 (2012).
5. Freeborough, P.A., Fox, N.C. The boundary shift integral: An accurate and robust measure of cerebral volume changes from registered repeat MRI. *IEEE transactions on medical imaging* 16(5), 623-629 (1997).
6. Wu, G.R., Wang, Q., Shen, D.G., NeuroImaging, A.D. Registration of longitudinal brain image sequences with implicit template and spatial-temporal heuristics. Neuroimage 59(1),404-421 (2012).
7. Staring, M., van der Heide, U.A., Klein, S., Viergever, M.A., Pluim, J.P. Registration of cervical MRI using multifeature mutual information. *IEEE transactions on medical imaging* 28(9),1412-1221 (2009).
8. Sonka, M. Image processing, analysis, and machine vision. *Thomson,* (2007).
9. Rueckert, D., Sonada, L.I., Hayes, C., Hill, D.L.G., Hawkes, D.J. Nonrigid registration using free-form deformations: applications to breast MR images. IEEE transactions on medical imaging 18(8), 712-721 (1999).
10. Ibanez, L., Schroeder, W., Ng, L., Consortium T.I.S., Hamming, R. The ITK software guide, Kitware, Inc., 2003.

## Supplement 2

Supplementary Movie 1-Segmentation and Quantification of Lungs from CT. Please click the link below (use google account if necessary):

<https://docs.google.com/open?id=0B2XUKmP9htKTUlIxUVJabzZtNjQ>

## Supplement 3

Supplementary Movie 2-Segmentation and Quantification of PET Images. Please click the link below (use google account if necessary):

<https://docs.google.com/open?id=0B2XUKmP9htKTYUNTRUt4UTFpdEU>

## Supplement 4

Supplementary Movie 3- Visualization of FDG uptake longitudinally in a rabbit model (rabbit #6). Please click the link below (use google account if necessary):

<https://docs.google.com/file/d/0B2XUKmP9htKTVTI4bFZ6NTJWTlk/edit?pli=1>

Supplementary Movie 4- Visualization of FDG uptake longitudinally in a rabbit model (rabbit #7). Please click the link below (use google account if necessary):

<https://docs.google.com/file/d/0B2XUKmP9htKTcE5EMUltbUpJd0E/edit?usp=sharing>

Supplementary Movie 5- Visualization of FDG uptake longitudinally in a ferret model (ferret #2214). Please click the link below (use google account if necessary):

[https://docs.google.com/file/d/0B2XUKmP9htKTVTI4bFZ6NTJWTlk/edit?pli=1](https://docs.google.com/file/d/0B2XUKmP9htKTaUkyQzVzbDZaenc/edit?usp=sharing)
